# Marine sentinels using eDNA to track *Physalia* sp. in the Gulf of Thailand

**DOI:** 10.1371/journal.pone.0326215

**Published:** 2025-06-24

**Authors:** Thanaporn Suebsuya, Panagiotis Madesis, Chatmongkon Suwannapoom, Maslin Osathanunkul

**Affiliations:** 1 Department of Biology, Faculty of Science, Chiang Mai University, Chiang Mai, Thailand; 2 Institute of Applied Biosciences, Centre for Research & Technology Hellas (CERTH), Thessaloniki, Greece; 3 Laboratory of Molecular Biology of Plants, Department of Agriculture, Crop Production and Rural Environment, University of Thessaly, Volos, Magnesia, Greece; 4 School of Agriculture and Natural Resources, University of Phayao, Muang District, Phayao, Thailand; Tsinghua University, CHINA

## Abstract

*Physalia* sp. is among the world’s most hazardous marine species, posing a significant threat to public safety and Thailand’s tourism sector. Traditional survey methods such as trawling and netting are time-consuming, potentially disruptive to marine ecosystems, and often lack the precision needed for effective monitoring. This study employed environmental DNA (eDNA) analysis to investigate the distribution of *Physalia* sp. across 45 sampling sites in eight provinces along the Gulf of Thailand. Using species-specific primers and probes targeting the COI region, we successfully detected *Physalia* sp. eDNA in four provinces: Chonburi, Rayong, Chumphon, and Songkhla. Notably, high eDNA concentrations were observed in Songkhla province, correlating with direct beach observations and public health warnings. The detection in Chumphon province represents a previously undocumented distribution area for this species in Thailand. Bayesian occupancy modeling revealed moderate true-positive detection rates for field samples (θ_11_ = 0.627) and high rates for qPCR replicates (p_11_ = 0.9), with notably low false-positive probabilities (θ_10_ = 0.008, p_10_ = 0.01), demonstrating the reliability of our eDNA-based approach. These findings demonstrate the utility of eDNA technology as a non-invasive, sensitive tool for monitoring hazardous marine species, with important implications for public safety and marine ecosystem management.

## Introduction

*Physalia* sp. is a hazardous marine organism belonging to the phylum Cnidaria. Although it resembles a jellyfish, it is actually classified under the class Hydrozoa and order Siphonophorae. It comprises only two species: *Physalia physalis* and *Physalia utriculus*. These organisms are commonly found in the Atlantic, Pacific, and Indian Oceans [1–3]. They consist of a pneumatophore, which serves as the above-water float with a diameter ranging from 3 to 12 inches, and long tentacles that extend up to 30 meters beneath the water’s surface. These tentacles contain thousands of venomous intracellular organelles capable of subduing prey and posing a threat to humans [1,4]. *Physalia* stings are known for their intense pain and systemic effects. They cause immediate local symptoms and severe pain. Moreover, there is a possibility that injuries to the skin could develop into necrotic conditions within 24 hours [2]. As a result of these characteristics, these siphonophore poses a significant danger to humans, having an impact on various countries, including Brazil, Australia, Portugal, Mexico, Indonesia, Thailand, and so forth [2,3,5]. In the context of Thailand, the Department of Marine and Coastal Resources (DMCR) has systematically recorded instances of *Physalia* sp. sightings since 2011. These surveys involve the use of trammel nets, surface gill nets, and beach seines, as well as on-foot exploration of the beach area. Additionally, they gather incident reports and receive specimen samples from fishermen or individuals in the area. These surveys are conducted four to ten times a year. Specifically, in the year 2021, these species manifestations were documented in diverse regions spanning Chonburi, Rayong, Phetchaburi, Prachuap Khiri Khan, Songkhla, Phuket, and Krabi provinces [6]. The comprehensive geographical distribution of these sightings underscores the pervasive presence of *Physalia* sp. within the marine ecosystems of Thailand during the specified timeframe.

Traditional methods for surveying jellyfish often yield inconsistent results, as certain species can only be effectively detected using specific types of fishing gear [7]. These limitations require the use of multiple sampling tools to achieve comprehensive monitoring, making the process time-consuming and heavily reliant on skilled personnel. As a result, there is an increasing need to develop and adopt alternative approaches that are not only accurate and efficient but also environmentally non-invasive.

Environmental DNA (eDNA) constitutes nuclear or mitochondrial DNA liberated into the environment through various biological processes, including secretions, skin cells, feces, urine, and mucus [8]. eDNA approach is a fast and accurate procedure that does not harm living species, therefore improving upon the limits of traditional survey methods. The approach is highly versatile and can be used to study a wide range of living organisms, including microorganisms such as fungi and bacteria [9], terrestrial and freshwater animals like the giant catfish (*Pangasianodon gigas*) [10] and even plant species like *Sapria himalayana*, which is a root parasitic plant [11]. Although eDNA analysis is commonly used in terrestrial and freshwater settings, it is also highly useful in marine environments, making it especially relevant for studying marine life.

In the study of various jellyfish species, eDNA-based detection has proven valuable, including the detection of box jellyfish species: *Chironex fleckeri*, *Copula sivickisi*, *Carybdea xaymacana*, *Carukia barnesi* and *Chiropsoides buitendijki* all of which are known for their lethal nature [12,13]. The research demonstrated the effectiveness of using eDNA technology, particularly in detecting both the medusa stage and the small polyps, which are challenging to survey through direct observation [12]. eDNA study has also been employed to estimate the biomass of various marine organisms, including fish and jellyfish [14,15]. Takahashi *et al*. (2020) demonstrated a positive correlation between eDNA concentrations and visually estimated biomass in both groups, with jellyfish exhibiting higher eDNA concentrations per biomass than fish [14]. Furthermore, Minamoto *et al*. (2017) showed that eDNA concentrations closely reflected the spatial and temporal abundance of *Chrysaora pacifica*, supporting the use of eDNA approach for assessing jellyfish distribution and relative biomass in marine environments [15].

The aforementioned studies employing the eDNA technique on various jellyfish species highlight a notable and significant gap in the literature regarding the application of eDNA analysis for monitoring venomous marine organism, particularly the *Physalia* species. Given the substantial risk posed by these siphonophore to both tourists and local inhabitants, it is imperative to ascertain its presence in marine environments. In Thailand, data on *Physalia* sp. is limited to records from the Department of Marine and Coastal Resources [6], which rely exclusively on traditional survey methods. This lack of advanced monitoring techniques underscores the need for more precise, innovative approaches, such as eDNA technique, to better understand *Physalia* sp. distribution and reduce risks to public health and marine ecosystems. Therefore, this research aims to investigate the distribution of *Physalia* sp. in the Gulf of Thailand using eDNA analysis, addressing the inherent limitations of conventional monitoring methods.

This work, by leveraging the innovative eDNA approach, would provide a more comprehensive and precise assessment of the presence of *Physalia* species. Conventional techniques for jellyfish identification, often hampered by logistical challenges and limited spatial-temporal accuracy, can significantly benefit from the sensitivity and non-invasive nature of eDNA analysis. This technique enables the detection of tiny quantities of DNA released by organisms into their surroundings, enabling prompt and precise identification of their existence without the necessity of directly observing or capturing them.

Understanding the spatial distribution and population dynamics of *Physalia* sp. is essential for mitigating the risks associated with their venomous stings, which can pose severe health threats to humans. The outcomes of this study are expected to enhance the management and safety protocols in coastal areas, contributing to the protection of both public health and the marine ecosystem. Moreover, this research could serve as a foundational framework for future studies on other venomous marine organisms, promoting the broader application of eDNA technology in marine biodiversity monitoring and conservation efforts.

## Materials and methods

### Ethics statement

The study was granted approval with protocol number 64 02 04 005 by the Institutional Ethical Committee of Animal Experimentation of the University of Phayao, Phayao, Thailand. All experiments were conducted in accordance with the relevant guidelines and regulations. The authors also confirm compliance with the ARRIVE guidelines.

### Sea water samples collection and DNA extraction

**Fig 1:**
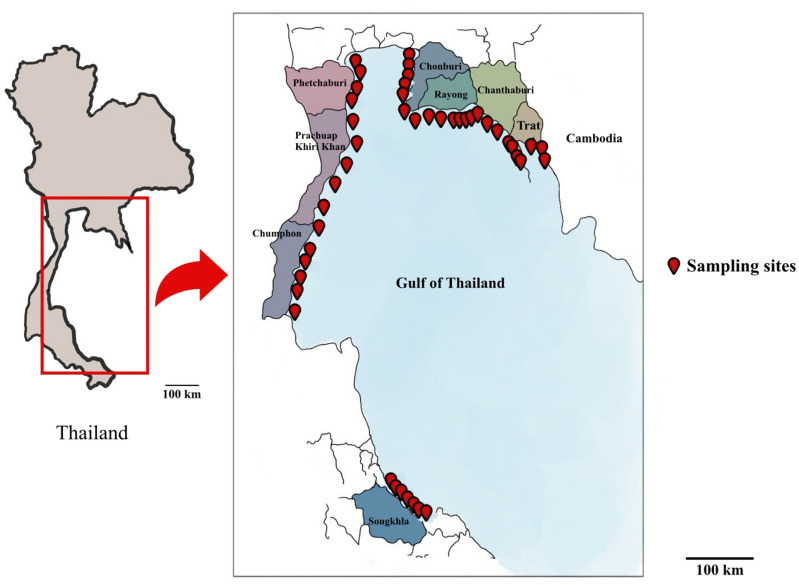
The sea water sampling sites across eight provinces. Red pins represent sampling sites.

In this study, the focus was on the Gulf of Thailand. The sampling sites were strategically distributed across eight provinces, namely Chonburi, Rayong, Chanthaburi, Trat, Phetchaburi, Prachuap Khiri Khan, Chumphon, and Songkhla (Fig 1). The coordinates for these locations are detailed in S1 Table. We conducted water sampling at 45 independent coastal locations across multiple provinces. Within each province, sampling sites were systematically selected to capture both broad-scale geographic variation and fine-scale spatial heterogeneity. The site coding system reflects this hierarchical sampling design: provincial prefixes (e.g., RY: Rayong, TR: Trat) indicate the primary geographic unit, while numerical suffixes denote discrete sampling locations. Sites with decimal extensions (e.g., RY1.1, RY1.2) represent independent sampling points within a continuous coastal segment, separated by 2–3 kilometers. At each site, three biological replicates (A, B, C) were collected and analyzed independently using qPCR to assess local detection consistency.

Our sampling design incorporated two levels of replication to assess both field-level and analytical variation. At each sampling site, we collected three independent 1,000 mL water samples (biological replicates A, B, and C), separated by approximately 10–20 meters to account for fine-scale spatial heterogeneity. Each biological replicate underwent separate DNA extraction and purification. Subsequently, each extracted DNA sample was analyzed in triplicate using qPCR (technical replicates) to assess analytical precision and reliability of detection. This hierarchical replication structure yielded nine total qPCR measurements per sampling site (3 biological replicates × 3 technical replicates), allowing robust assessment of both field-level variation and analytical reproducibility.

To ensure the integrity of the samples and avoid contamination, rigorous sterilization methods were employed for all field equipment, utilizing 10% bleach, UV-Crosslinker, or autoclaving before use. Field sampling was conducted in publicly accessible coastal areas. We have confirmed that all sampling activities complied with local regulations and did not involve restricted or protected areas.

At each site, sea water samples of 1,000 mL were collected and filtered through a 0.7 μm pore size glass microfiber filter (Whatman International Ltd., Maidstone, UK). Subsequent to filtration, DNA extraction from the filter paper was carried out using the Qiagen DNeasy Blood and Tissue Kit (Qiagen, Hilden, Germany), following the manufacturer’s protocol with a slight modification. Specifically, DNA from all samples was eluted twice with 75 μl AE buffer, resulting in a total volume of 150 μl, aimed at obtaining a more concentrated eDNA solution. The amounts used were 360 μl of ATL buffer, 40 μl of Proteinase K, 400 μl of AL buffer, and 400 μl of Ethanol. (following the protocol outlined by Osathanunkul & Minamoto) [16].

To eliminate any PCR inhibitors, the samples were processed with the OneStep PCR Inhibitor Removal Kit (Zymo Research). The effectiveness of inhibition removal was verified using internal controls targeting the COI gene of *Garra cambodgiensis*, a freshwater species absents from marine environments [17]. The ΔCq value for these controls across all samples was less than 3 cycles, indicating minimal PCR inhibition [18].

### Development of primers and probe

Species-specific primers and probes for *Physalia* sp. were designed by analyzing sequences from four DNA regions (COI, 16S rRNA, 18S rRNA, and 28S rRNA) obtained from GenBank (see S2 Table). The design process utilized two complementary bioinformatics tools: NCBI Primer–BLAST (https://blast.ncbi.nlm.nih.gov/Blast.cgi) and PrimerQuest™ (https://eu.idtdna.com/pages/tools/primerquest). After comparative analysis of all four regions, the COI region was selected as the optimal target for species-specific detection. Primers and probe designed specifically for *Physalia* sp. were used in the qPCR experiments, which amplified an 82-bp region of the COI gene (as shown in Table 1).

**Table 1:** The information of COI primers and probe.

Name	Type	Sequences (5’-3’)	GC content (%)	Melting Temperature (°C)	Length (bp)
PhysaliaCOI-F409	Forward primer	5’-GGGGATCAGTTGATATGGC-3’	52.6	52.8	19
PhysaliaCOI-R468	Reverse primer	5’-GTGGTTATGAAGTTAATAGCACC-3’	39.1	51.5	23
PhysaliaCOI-P436	Probe	5’-/56-FAM/GTTTACACTGTGCGGGTGC/3MGB-NFQ/ −3’	57.9	57	19

The sequences are displayed in 5’ end to 3’ end direction, with the GC content shown as a percentage, melting point indicated in degrees Celsius, and length represented by the number of base pairs.

### Specificity and sensitivity test

Primer and probe specificity was validated through both *in silico* and *in vitro* approaches. Initial *in silico* validation was performed using BLASTn (Basic Local Alignment Search Tool) against the NCBI database and our own sequences of four morphotype specimens collected from the studied area, which were obtained from the DMCR (see S2 Table for COI sequences). Subsequent *in vitro* testing employed PCR and qPCR analyses using DNA extracted from co-occurring species, including *Chironex indrasaksajiae*, *Pelagia* sp., *Morbakka* sp., *Lobonemoides robustus*, *Lobonema smithii*, *Copula sivickisi*, *Meteorona* sp., and *Chiropsoides buitendijki* [19], as well as four morphotypes of *Physalia* found in the Thai coastal region.

Assay sensitivity was evaluated using a 10-fold serial dilution series (3 × 10⁸ to 3 × 10 ⁻ ¹ copies/reaction) generated from a synthetic DNA gBlock® Gene Fragment (IDT, Coralville, CA). DNA concentrations were verified using a Qubit 4.0 Fluorometer (Thermo Fisher Scientific). Twelve replicates were analyzed at each dilution level to determine the limit of detection (LOD) and limit of quantification (LOQ) using the R script developed by Klymus *et al*. 2020 [20]. Samples were considered positive when amplification exceeded the Cq threshold in any replicate.

### qPCR analysis

All extracted eDNA was analyzed with qPCR using the designed primers and probe, which have been confirmed to be specific only to *Physalia* sp. The qPCR was conducted using the Rotor-Gene Q thermocycler (Qiagen, Hilden, Germany). The qPCR amplifications for all eDNA samples were conducted in three replicates, following the protocol outlined by Osathanunkul, 2022 [10]. This involved a final volume of 20 μl, comprising 10.0 μl of 2 × TaqMan Environmental Master Mix 2.0 (Thermo Fisher Scientific), 2.0 μl of DNA template, 900 nM each of the forward and reverse primer, and 125 nM of the probe. The samples were run through a series of conditions: an initial 10-minute incubation at 95 °C, followed by 50 cycles involving denaturation at 95 °C for 30 second and annealing/extension at 58.8 °C for 1 minute. In this study, the positive control employed was the DNA extract from a *Physalia* sp. fragment, while the negative control involved all PCR reagents without any DNA.

### Statistical analysis

Species occurrence patterns were analyzed using Bayesian occupancy modeling implemented through the RShiny application ‘eDNA 1.0’ [21]. This hierarchical modeling approach accounts for imperfect detection at two levels: (1) the field sampling stage, which represents the probability of detecting target DNA in environmental samples, and (2) the laboratory analysis stage, which represents the probability of successful qPCR amplification given the presence of target DNA.

The model estimated several key parameters. Species occurrence probability (ψ) represents the probability that *Physalia* sp. is present at a given site. Field-level detection probabilities include the true-positive detection rate (θ_11_), which represents the probability of detecting *Physalia* sp. eDNA when it is present, and the false-positive detection rate (θ_10_), which accounts for cases where eDNA is detected despite the absence of *Physalia* sp. The false-negative probability (1 − θ_11_) represents the likelihood of failing to detect *Physalia* sp. eDNA despite its presence in the sample. Similarly, laboratory-level detection probabilities include the true-positive rate for qPCR replicates (p_11_) (successful amplification when target DNA is present), the false-positive rate for qPCR replicates (p_10_) (incorrect detection due to non-target amplification), and the false-negative probability for qPCR replicates (1 − p_11_) (failure to amplify target DNA despite its presence). The analysis was conducted using default model settings with 2,000 burn-in iterations followed by 2,000 main iterations across four chains. Data were pooled across sampling sites and assays to ensure robust sample sizes. Model parameters were adjusted following recommendations by Diana *et al*. 2021 to optimize detection accuracy [21].

## Results and discussion

### Development of primers and probe

The development of a species-specific qPCR assay required systematic evaluation of four distinct genetic markers: cytochrome c oxidase subunit I (COI), 16S rRNA, 18S rRNA, and 28S rRNA. After comparative analysis, the mitochondrial COI gene emerged as the optimal target for specific detection. While the ribosomal RNA genes (16S, 18S, and 28S) showed high conservation across related species, the COI region provided sufficient sequence variation to ensure species specificity. This selection was based on multiple criteria: the presence of conserved regions suitable for primer binding, sequence divergence between target and related species, and appropriate amplicon length for qPCR efficiency. The designed primer pair and probe targeting the COI region (Table 1) demonstrated high specificity when tested against reference sequences *in silico*.

The success of eDNA detection heavily relies on the design and validation of species-specific molecular markers [22,23]. Our primers were strategically designed with optimal lengths (19 and 23 base pairs) to balance amplification efficiency with targeting specificity [24,25]. These relatively short primers offer several technical advantages: they minimize potential secondary structure formation that could impede amplification, reduce the likelihood of non-specific binding, and facilitate efficient target DNA amplification under standard qPCR conditions [25].

### Specificity and sensitivity test

The primers and probe underwent rigorous validation to ensure target species specificity through both *in silico* and *in vitro* approaches. Initial *in silico* validation was conducted using BLASTn (Basic Local Alignment Search Tool) to evaluate sequence homology and potential cross-reactivity with related species. The BLASTn analysis confirmed that the selected primer and probe sequences aligned exclusively with the target species’ COI region.

Following the computational validation, *in vitro* testing was performed using conventional PCR and qPCR analyses. These experimental validations confirmed the primers’ ability to amplify the target region specifically and the probe’s capacity to generate fluorescent signals only in the presence of target DNA. This validation strategy, integrating both computational prediction and laboratory testing, established the reliability and specificity of the designed primers and probe for subsequent eDNA detection via qPCR analysis.

The robust validation of our molecular markers, combining both *in silico* and *in vitro* approaches, established their reliability for specific detection of *Physalia* sp. DNA in marine samples. By targeting well-characterized DNA regions and confirming specificity through multiple validation steps, we minimized the risk of false-positive detections while maintaining high analytical sensitivity [26]. The development of this reliable detection system provides a foundation for implementing routine monitoring programs that could help protect public health through early warning systems and informed decision-making about beach safety measures.

The limit of detection (LOD) and limit of quantification (LOQ) were determined through analysis of a 10-fold serial dilution series (3 × 10⁸ to 3 × 10 ⁻ ¹ copies/reaction) of synthetic Physalia sp. DNA standards. Each concentration was analyzed in 12 technical replicates across three independent qPCR runs. The LOD was established as the lowest concentration yielding positive amplification in ≥95% of replicates (2.07 copies/reaction, mean Cq = 37.92). The LOQ was defined as the lowest concentration that could be reliably quantified with a coefficient of variation <35% (2.07 copies/reaction, mean Cq = 37.92). Standard curves demonstrated high amplification efficiency (99.03%) and strong linearity (R² = 0.9978) across the quantifiable range.

Following established eDNA detection protocols, we classified amplifications based on empirically determined thresholds: positive detections (Cq ≤ 37.92, corresponding to ≥ 2.07 copies/reaction), below quantification limit (Cq ≥ 37.93 and < 45), and non-detection (Cq ≥ 45 or no amplification). This approach yielded 12 positive detections, 8 samples below the quantification limit, and 25 non-detections across our 45 sampling sites, ensuring robust and reliable presence/absence determination for *Physalia* sp.

### qPCR analysis

Quantitative PCR analysis revealed *Physalia* sp. eDNA presence across the sampling region, with positive detections at 20 of 45 sites (44.4%) spanning four coastal provinces. Of these, 12 sites yielded quantifiable eDNA concentrations: Chonburi (2 sites), Rayong (2 sites), Chumphon (1 site), and Songkhla (7 sites) (see Table 2). Eight additional sites showed detectable but non-quantifiable levels (below quantification limit, bq), while the remaining 25 sites showed no detectable eDNA (nd). Complete site-specific detection data and corresponding eDNA concentrations are provided in Supplementary S3 Table.

**Table 2:** eDNA detection for each sampled site.

Sampled sites ID	Provinces	eDNA detection	Sampled sites ID	Provinces	eDNA detection
CB1	Chonburi	Positive	PCK1	Prachuap Khiri Khan	Negative
CB2		Negative	PCK2.5		Negative
CB3		Negative	PCK4		Negative
CB4		Negative	PCK5.5		Negative
CB5		Negative	PCK7		Negative
CB6		Positive	CP1	Chumphon	Negative
CB7		Negative	CP3		Negative
RY1	Rayong	Negative	CP4		Positive
RY1.1		Negative	CP5		Negative
RY1.2		Negative	CP6		Negative
RY2		Positive	CP8		Negative
RY3		Negative	PB1	Phetchaburi	Negative
RY4		Negative	PB2		Negative
RY5		Positive	PB3		Negative
JT1	Chanthaburi	Negative	PB3.55		Negative
JT2		Negative	SK1	Songkhla	Positive
TR1	Trat	Negative	SK2		Positive
TR2		Negative	SK3		Positive
TR3		Negative	SK4		Positive
TR5		Negative	SK5		Positive
TR5n		Negative	SK6		Positive
TR6		Negative	SK7		Positive

For eDNA detection, there are two categories: (1) Positive and (2) Negative.

**Fig 2:**
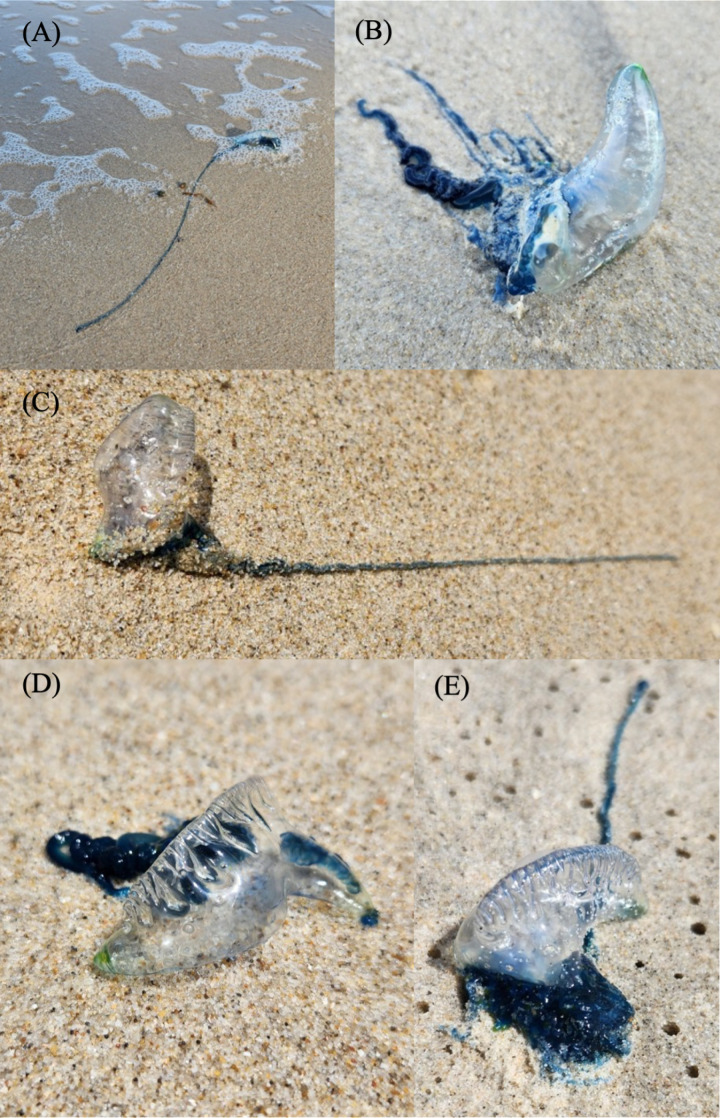
The *Physalia* sp. observed on Songkhla Province beaches during the seawater sampling. (A) represents *Physalia* sp. found at SK2, (B) at SK3, (C) at SK4, (D) at SK5 and (E) at SK6.

*Physalia* sp. individuals were seen in large numbers on the beaches in Songkhla Province during the water collection (Fig 2), establishing an expectation for positive results in the subsequent eDNA study. The eDNA results indeed affirmed this anticipation, revealing unequivocally positive outcomes across all seven scrutinized locations in Songkhla Province. Remarkably, three of these sites revealed remarkably high amounts of *Physalia* sp. eDNA, measuring at SK3: 10,545 copies/mL, SK4: 55,967 copies/mL and SK5: 73,704 copies/mL (S3 Table).

The qPCR analysis revealed *Physalia* sp. eDNA presence in 12 of 45 sampling sites across four provinces: Chonburi (CB1, CB6), Rayong (RY2, RY5), Chumphon (CP4), and Songkhla (SK1-SK7). The detection in Chumphon province is particularly significant as it represents a previously undocumented distribution area for this species in Thailand. Notably, in Songkhla province, *Physalia* sp. eDNA was detected at all sampling points, with several locations showing high concentrations that corresponded with direct visual observations of numerous specimens on beaches during sampling (Fig 2). This correlation between eDNA concentrations and observed abundance aligns with previous studies suggesting that eDNA levels can serve as indicators of species biomass or density in marine environments [27–29].

The high abundance of *Physalia* sp. observed in Songkhla Province coincided with official public health warnings about the presence of these potentially dangerous organisms in coastal waters. Local authorities attributed this congregation to the strong northeastern monsoon’s influence on sea currents and wave patterns [30]. This observation is consistent with known *Physalia* sp. behavior, as these organisms have limited self-propulsion capabilities and rely primarily on wind and ocean currents for their movement and distribution [31]. This passive dispersal mechanism likely explains their patchy distribution pattern across the sampling sites.

In Phetchaburi province, while no positive detections were recorded, three of four sites (PB2, PB3, and PB3.55) yielded results categorized as below the limit of quantification (bq). In qPCR-based eDNA analysis, negative results are typically classified into two categories: no detection (nd), where no eDNA amplification occurs, and below quantification (bq), where minimal eDNA amplification is observed but falls below the established limit of quantification (LOQ). This distinction is crucial as bq results suggest potential species presence, albeit at very low concentrations. Such classification protocols are standard in eDNA studies across various marine and freshwater organisms [20,32]. Recent advancements in eDNA analysis have significantly enhanced detection capabilities.

### Occupancy probabilities

At the environmental sample level, the model estimated a true-positive detection probability (θ_11_) of 0.627, with a corresponding false-negative rate (1 − θ_11_) of 0.373. The false-positive probability for environmental samples (θ_10_) was notably low at 0.008, indicating high sampling reliability.

Analysis of qPCR technical replicates demonstrated robust analytical performance, with a high true-positive detection probability (p_11_) of 0.9 and consequently a low false-negative rate (1 − p_11_) of 0.1. The false-positive probability for qPCR replicates (p_10_) was minimal at 0.01, suggesting high analytical specificity. All occupancy and detection probability parameters are summarized in Table 3.

**Table 3:** Summaries of the probability parameters in both field sampling stage and laboratory stage.

Parameter	Mean
Sample true-positive probability (θ_11_)	0.627
Sample false-positive probability (θ_10_)	0.008
qPCR replicate true-positive probability (p_11_)	0.900
qPCR replicate false-positive probability (p_10_)	0.010
Sample false-negative probability (1-θ_11_)	0.373
qPCR false-negative probability (1- p_11_)	0.100

Our occupancy modeling revealed complex patterns of detection probability across different analytical levels. The true-positive detection probability for field-collected samples (θ_11_ = 0.627) indicates moderate success in detecting *Physalia* sp. DNA from environmental samples. At the laboratory level, the true-positive rate for qPCR replicates (p_11_ = 0.9) demonstrating robust analytical performance for samples containing detectable DNA.

The assay exhibited high specificity, with false-positive probabilities remaining remarkably low for both field samples (θ_10_ = 0.008) and qPCR replicates (p_10_ = 0.01), supporting the reliability of the method in minimizing non-target DNA detection [33]. The contrast between field sample false-negative probability (1 − θ_11_ = 0.373) and qPCR replicate false-negative probability (1 − p_11_ = 0.1) reveals a key methodological consideration: while the assay performs well under controlled laboratory conditions, the higher field-level false-negative rate suggests opportunities for optimizing sampling protocols to enhance detection efficiency [34]. This observation aligns with findings from Osathanunkul 2024, which highlighted the importance of refined field sampling strategies for jellyfish eDNA detection [13].

## Conclusions

Our eDNA-based investigation revealed novel distribution patterns of *Physalia* sp. across the Gulf of Thailand, including its presence in previously undocumented regions. The molecular detection method proved highly specific and reliable, though differences between laboratory and field detection rates suggest opportunities for methodological refinement. These findings have direct implications for public safety, as understanding the spatial distribution of this venomous siphonophore enables more effective early warning systems. The successful implementation of eDNA monitoring for *Physalia* sp. provides a framework for broader marine surveillance programs and demonstrates how molecular approaches can enhance both public safety and marine ecosystem management in coastal waters.

## Supporting information

S1 Table Details of sea water sampling sites across 8 provinces in this study. (DOCX)

S2 Table The sequences information retrieved from the National Center for Biotechnology Information (NCBI). (DOCX)

S3 Table qPCR results for each sampled site, where Cq refers to the quantification cycle. For qPCR results, there are three categories: Positive (+), below the limit of quantification (bq: Cq = 37.93–44.99) and non-detect (nd: Cq ≥ 45 or No amplification). In positive qPCR results, eDNA concentration is expressed in copies/mL. The reported average Cq value represents the mean Cq across biological replicates A, B, and C.
(DOCX)
