# Supplementary material for: Marine sentinels using eDNA to track Physalia sp. in the Gulf of Thailand
Source: PLoS One. 2025 Jun 24;20(6):e0326215. doi: 10.1371/journal.pone.0326215 (PMC12186917; doi:10.1371/journal.pone.0326215)
Supplement: S1 Table — (DOCX) [file pone.0326215.s001.docx]

**Supplementary Information for**

Marine Sentinels Using eDNA to Track *Physalia* sp. in the Gulf of Thailand

Thanaporn Suebsuya^1^, Panagiotis Madesis^2,3^, Chatmongkon Suwannapoom^4^ and Maslin Osathanunkul^1*^

^1^ Department of Biology, Faculty of Science, Chiang Mai University, Chiang Mai, Thailand
^2^ Institute of Applied Biosciences, Centre for Research & Technology Hellas (CERTH), Thessaloniki, Greece
^3^ Laboratory of Molecular Biology of Plants, Department of Agriculture, Crop Production and Rural Environment, University of Thessaly, Volos, Magnesia, Greece
^4^ School of Agriculture and Natural Resources, University of Phayao, Muang District, Phayao, Thailand

**S1 Table.** Details of sea water sampling sites across 8 provinces in this study.

| **ID** | **Provinces** | **Coordinates** | **ID** | **Provinces** | **Coordinates** |
| --- | --- | --- | --- | --- | --- |
| CB1 | Chonburi | 12.60296, 100.94418 | PB1 | Phetchaburi | 13.26520, 99.94187 |
| CB2 |  | 12.74378, 100.84120 | PB2 |  | 13.00604, 100.06607 |
| CB3 |  | 12.88538, 100.87570 | PB3 |  | 12.80188, 99.98691 |
| CB4 |  | 12.99208, 100.92132 | PB3.55 |  | 12.67374, 99.96056 |
| CB5 |  | 13.07886, 100.88149 | PCK1 | Prachuap Khiri Khan | 12.56944, 99.96263 |
| CB6 |  | 13.17441, 100.91901 | PCK2.5 |  | 12.24298, 99.98451 |
| CB7 |  | 13.28327, 100.91469 | PCK4 |  | 11.81935, 99.79961 |
| RY1 | Rayong | 12.70071, 101.71548 | PCK5.5 |  | 11.49941, 99.63238 |
| RY1.1 |  | 12.64396, 101.64634 | PCK7 |  | 11.16559, 99.49695 |
| RY1.2 |  | 12.64905, 101.62057 | CP1 | Chumphon | 10.92799, 99.49374 |
| RY2 |  | 12.63152, 101.56408 | CP3 |  | 10.56179, 99.27378 |
| RY3 |  | 12.61010, 101.38458 | CP4 |  | 10.42545, 99.26711 |
| RY4 |  | 12.66504, 101.23332 | CP5 |  | 10.32880, 99.15311 |
| RY5 |  | 12.67462, 101.06729 | CP6 |  | 10.19262, 99.18554 |
| JT1 | Chanthaburi | 12.47924, 102.06471 | CP8 |  | 9.79298, 99.14032 |
| JT2 |  | 12.53307, 101.94257 | SK1 | Songkhla | 7.77884, 100.36879 |
| TR1 | Trat | 11.71762, 102.90369 | SK2 |  | 7.64126, 100.40360 |
| TR2 |  | 11.94409, 102.78446 | SK3 |  | 7.47484, 100.44566 |
| TR3 |  | 12.15272, 102.62399 | SK4 |  | 7.35321, 100.49079 |
| TR5 |  | 12.16894, 102.40630 | SK5 |  | 7.21516, 100.59532 |
| TR5n |  | 12.16705, 102.39003 | SK6 |  | 7.02637, 100.72863 |
| TR6 |  | 12.27428, 102.26547 | SK7 |  | 6.96002, 100.84651 |
| TR6.5 |  | 12.12130, 102.16500 |  |  |  |
