# Supplementary material for: Marine sentinels using eDNA to track Physalia sp. in the Gulf of Thailand
Source: PLoS One. 2025 Jun 24;20(6):e0326215. doi: 10.1371/journal.pone.0326215 (PMC12186917; doi:10.1371/journal.pone.0326215)
Supplement: S2 Table — (DOCX) [file pone.0326215.s002.docx]

**Supplementary Information for**

Marine Sentinels Using eDNA to Track *Physalia* sp. in the Gulf of Thailand

Thanaporn Suebsuya^1^, Panagiotis Madesis^2,3^, Chatmongkon Suwannapoom^4^ and Maslin Osathanunkul^1*^

^1^ Department of Biology, Faculty of Science, Chiang Mai University, Chiang Mai, Thailand
^2^ Institute of Applied Biosciences, Centre for Research & Technology Hellas (CERTH), Thessaloniki, Greece
^3^ Laboratory of Molecular Biology of Plants, Department of Agriculture, Crop Production and Rural Environment, University of Thessaly, Volos, Magnesia, Greece
^4^ School of Agriculture and Natural Resources, University of Phayao, Muang District, Phayao, Thailand

**S2 Table.** The sequences information retrieved from the National Center for Biotechnology Information (NCBI).

| **DNA regions** | **Spceies** | **Acession no.** |
| --- | --- | --- |
| **COI** | *Physalia* sp. | GQ120034 |
|  | *Physalia physalis* | MK084614 |
|  | *Chironex indrasaksajiae* | KT223648 |
|  | *Pelagia* sp. | KY611299 |
|  | *Morbakka* sp. | JN202985 |
|  | *Lobonemoides robustus* | JN203013 |
|  | *Lobonema smithii* | KY610574 |
|  | *Copula sivickisi* | LC726368 |
|  | *Meteorona kishinouyei* | LC726365 |
|  | *Alatina morandinii* | LC726366 |
|  | *Chironex* sp. | MG544096 |
|  | *Cassiopea ornata* | LC198737 |
|  | *Acromitus flagellatus* | OR230087 |
|  | *Catostylus townsendi* | MN395693 |
|  | *Phyllorhiza pacifica* | MN395673 |
|  | *Phyllorhiza punctata* | GQ120101 |
|  | *Chrysaora chinensis* | MF141605 |
|  | *Rhopilema hispidum* | JN203011 |
|  | *Aurelia* sp. | EU010386 |
|  | *Versuriga anadyomene* | KX904853 |
|  | *Aequorea* sp. | JQ716188 |
|  | *Porpita porpita* | GQ120044 |
|  | *Velella velella* | MN107376 |

**S2 Table. (cont.)** The sequences information retrieved from the National Center for Biotechnology Information (NCBI).

| **DNA regions** | **Spceies** | **Acession no.** |
| --- | --- | --- |
| **16S rRNA** | *Physalia utriculus* | AY512511 |
|  | *Physalia physalis* | AY935284 |
|  | *Chironex indrasaksajiae* | KX090147 |
|  | *Pelagia* sp. | KY610733 |
|  | *Lobonema smithii* | KY610580 |
|  | *Copula sivickisi* | GQ849113 |
|  | *Chiropsoides buitendijki* | KY980652 |
|  | *Tripedalia cystophora* | L10829 |
|  | *Acromitus flagellatus* | JN202936 |
|  | *Catostylus townsendi* | KY610587 |
|  | *Phyllorhiza pacifica* | KY610623 |
|  | *Phyllorhiza punctata* | JX393272 |
|  | *Chrysaora chinensis* | MF141691 |
|  | *Rhopilema hispidum* | AB720917 |
|  | *Aurelia* sp. | MF981181 |
|  | *Versuriga anadyomene* | KX904852 |
|  | *Aequorea* sp. | KY363940 |
|  | *Porpita porpita* | AY935322 |
|  | *Velella velella* | EU305487 |

**S2 Table. (cont.)** The sequences information retrieved from the National Center for Biotechnology Information (NCBI).

| **DNA regions** | **Spceies** | **Acession no.** |
| --- | --- | --- |
| **18S rRNA** | *Physalia* sp. | FJ847276 |
|  | *Chironex indrasaksajiae* | KU097000 |
|  | *Pelagia* sp. | KY610847 |
|  | *Lobonema smithii* | KY610750 |
|  | *Copula sivickisi* | GQ849087 |
|  | *Meteorona kishinouyei* | LC033479 |
|  | *Chiropsoides buitendijki* | KY980654 |
|  | *Alatina morandinii* | LC047805 |
|  | *Tripedalia binata* | LC480260 |
|  | *Tripedalia cystophora* | GQ849088 |
|  | *Cassiopea ornata* | HM194785 |
|  | *Acromitus flagellatus* | KY610747 |
|  | *Catostylus townsendi* | KY610756 |
|  | *Phyllorhiza pacifica* | KY610774 |
|  | *Phyllorhiza punctata* | HG931673 |
|  | *Chrysaora chinensis* | OQ842252 |
|  | *Rhopilema hispidum* | KY610856 |
|  | *Aurelia* sp. | AY319851 |
|  | *Versuriga anadyomene* | KX904851 |
|  | *Aequorea* sp. | KY363972 |
|  | *Porpita porpita* | GQ424319 |
|  | *Velella velella* | EU876576 |

**S2 Table. (cont.)** The sequences information retrieved from the National Center for Biotechnology Information (NCBI).

| **DNA regions** | **Spceies** | **Acession no.** |
| --- | --- | --- |
| **28S rRNA** | *Physalia physalis* | EU448095 |
|  | *Pelagia* sp. | KY610989 |
|  | *Lobonema smithii* | KY610883 |
|  | *Copula sivickisi* | GQ849064 |
|  | *Tripedalia cystophora* | GQ849065 |
|  | *Cassiopea ornata* | HM194838 |
|  | *Acromitus flagellatus* | KY610880 |
|  | *Catostylus townsendi* | KY610919 |
|  | *Phyllorhiza pacifica* | KY610998 |
|  | *Phyllorhiza punctata* | KY611001 |
|  | *Chrysaora chinensis* | KY610955 |
|  | *Rhopilema hispidum* | ON427567 |
|  | *Aurelia* sp. | KY610901 |
|  | *Versuriga anadyomene* | HM194837 |
|  | *Porpita porpita* | EU883551 |
|  | *Velella velella* | EU879949 |
