# Supplementary material for: Marine sentinels using eDNA to track Physalia sp. in the Gulf of Thailand
Source: PLoS One. 2025 Jun 24;20(6):e0326215. doi: 10.1371/journal.pone.0326215 (PMC12186917; doi:10.1371/journal.pone.0326215)
Supplement: S3 Table — For qPCR results, there are three categories: Positive (+), below the limit of quantification (bq: Cq = 37.93–44.99) and non-detect (nd: Cq ≥ 45 or No amplification). In positive qPCR results, eDNA concentration is expressed in copies/mL. The reported average Cq value represents the mean Cq across biological replicates A, B, and C. (DOCX) [file pone.0326215.s003.docx]

**Supplementary Information for**

Marine Sentinels Using eDNA to Track *Physalia* sp. in the Gulf of Thailand

Thanaporn Suebsuya^1^, Panagiotis Madesis^2,3^, Chatmongkon Suwannapoom^4^ and Maslin Osathanunkul^1*^

^1^ Department of Biology, Faculty of Science, Chiang Mai University, Chiang Mai, Thailand
^2^ Institute of Applied Biosciences, Centre for Research & Technology Hellas (CERTH), Thessaloniki, Greece
^3^ Laboratory of Molecular Biology of Plants, Department of Agriculture, Crop Production and Rural Environment, University of Thessaly, Volos, Magnesia, Greece
^4^ School of Agriculture and Natural Resources, University of Phayao, Muang District, Phayao, Thailand

**S3 Table. qPCR results for each sampled site, where Cq refers to the quantification cycle.** For qPCR results, there are three categories: Positive (+), below the limit of quantification
(bq: Cq = 37.93-44.99) and non-detect (nd: Cq ≥ 45 or No amplification). In positive qPCR results, eDNA concentration is expressed in copies/mL. The reported average Cq value represents the mean Cq across biological replicates A, B, and C.

| **Sampled sites ID** | **Provinces** | **Cq average** | | | | **qPCR results** | **eDNA detection** | **eDNA concentration (copies/mL)** |
| --- | --- | --- | --- | --- | --- | --- | --- | --- |
|  |  | **A** | **B** | **C** | **Report** |  |  |  |
| CB1 | Chonburi | 0 | 39.88 | 37.18 | 37.18 | + | Positive | 0.534 |
| CB2 |  | 48.14 | 0 | 40.97 | 0 | nd | - | - |
| CB3 |  | 48.80 | 0 | 0 | 0 | nd | - | - |
| CB4 |  | 41.53 | 0 | 45.37 | 43.45 | bq | - | - |
| CB5 |  | 0 | 0 | 0 | 0 | nd | - | - |
| CB6 |  | 0 | 0 | 37.32 | 37.32 | + | Positive | 0.384 |
| CB7 |  | 0 | 0 | 0 | 0 | nd | - | - |
| RY1 | Rayong | 49.73 | 44.50 | 40.56 | 42.53 | bq | - | - |
| RY1.1 |  | 0 | 40.09 | 39.56 | 39.83 | bq | - | - |
| RY1.2 |  | 44.2 | 41.29 | 0 | 42.75 | bq | - | - |
| RY2 |  | 36.57 | 36.77 | 41.15 | 36.67 | + | Positive | 1.289 |
| RY3 |  | 47.68 | 0 | 0.00 | 0 | nd | - | - |
| RY4 |  | 0 | 40.47 | 46.47 | 0 | nd | - | - |
| RY5 |  | 35.21 | 38.76 | 36.65 | 35.93 | + | Positive | 2.352 |
| JT1 | Chanthaburi | 49.17 | 0 | 0 | 0 | nd | - | - |
| JT2 |  | 0 | 0 | 43.69 | 0 | nd | - | - |
| TR1 | Trat | 48.09 | 0 | 0 | 0 | nd | - | - |
| TR2 |  | 0 | 0 | 0 | 0 | nd | - | - |
| TR3 |  | 0 | 0 | 0 | 0 | nd | - | - |
| TR5 |  | 0 | 0 | 0 | 0 | nd | - | - |
| TR5n |  | 0 | 43.46 | 0 | 0 | nd | - | - |
| TR6 |  | 0 | 0 | 0 | 0 | nd | - | - |
| TR6.5 |  | 0 | 0 | 0 | 0 | nd | - | - |
| PB1 | Phetchaburi | 0 | 0 | 0 | 0 | nd | - | - |
| PB2 |  | 39.32 | 0 | 0 | 39.32 | bq | - | - |
| PB3 |  | 43.90 | 38.19 | 46.43 | 41.05 | bq | - | - |
| PB3.55 |  | 44.71 | 41.99 | 46.57 | 43.35 | bq | - | - |
| PCK1 | Prachuap  Khiri Khan | 0 | 41.36 | 49.37 | 0 | nd | - | - |
| PCK2.5 |  | 0 | 0 | 0 | 0 | nd | - | - |
| PCK4 |  | 0 | 0 | 0 | 0 | nd | - | - |
| PCK5.5 |  | 0 | 48.12 | 42.96 | 0 | nd | - | - |
| PCK7 |  | 0 | 0 | 48.73 | 0 | nd | - | - |
| CP1 | Chumphon | 0 | 0 | 45.82 | 0 | nd | - | - |
| CP3 |  | 0 | 40.07 | 0 | 0 | nd | - | - |
| CP4 |  | 36.84 | 37.11 | 35.63 | 36.53 | + | Positive | 1.554 |
| CP5 |  | 49.44 | 47.11 | 0 | 0 | nd | - | - |
| CP6 |  | 38.78 | 0 | 49.83 | 38.78 | bq | - | - |
| CP8 |  | 42.97 | 48.12 | 46.69 | 0 | nd | - | - |

**S3 Table. (cont.)** **qPCR results for each sampled site, where Cq refers to the quantification cycle.** For qPCR results, there are three categories: Positive (+), below the limit of quantification (bq: Cq = 37.93-44.99) and non-detect (nd: Cq ≥ 45 or No amplification). In positive qPCR results, eDNA concentration is expressed in copies/mL. The reported average Cq value represents the mean Cq across biological replicates A, B, and C.

| **Sampled sites ID** | **Provinces** | **Cq average** | | | | **qPCR results** | **eDNA detection** | **eDNA concentration (copies/mL)** |
| --- | --- | --- | --- | --- | --- | --- | --- | --- |
|  |  | **A** | **B** | **C** | **Report** |  |  |  |
| SK1 | Songkhla | 33.94 | 33.37 | 33.25 | 33.52 | + | Positive | 1.158 |
| SK2 |  | 33.37 | 33.81 | 32.44 | 33.21 | + | Positive | 1.396 |
| SK3 |  | 19.57 | 19.83 | 19.68 | 19.69 | + | Positive | 10,545.812 |
| SK4 |  | 17.23 | 17.11 | 17.19 | 17.18 | + | Positive | 55,967.228 |
| SK5 |  | 16.84 | 16.71 | 16.74 | 16.76 | + | Positive | 73,704.093 |
| SK6 |  | 31.73 | 32.11 | 32.38 | 32.07 | + | Positive | 2.895 |
| SK7 |  | 33.33 | 34.28 | 34.98 | 34.20 | + | Positive | 0.780 |
